# Supplementary material for: Multi‐dataset identification of innovative feature genes and molecular mechanisms in keratoconus
Source: J Cell Mol Med. 2024 Sep 19;28(18):e70079. doi: 10.1111/jcmm.70079 (PMC11412914; doi:10.1111/jcmm.70079)
Supplement: Supplementary file 2 — Material S2. [file JCMM-28-e70079-s002.zip › 7.GSEA analysis/2.ATOH7/KEGG_HUNTINGTONS_DISEASE.html]

Details for gene set KEGG\_HUNTINGTONS\_DISEASE[GSEA]

|  || Dataset | ATOH7.group.cls#h\_versus\_l |
| Phenotype | group.cls#h\_versus\_l |
| Upregulated in class | h |
| GeneSet | KEGG\_HUNTINGTONS\_DISEASE |
| Enrichment Score (ES) | 0.25773862 |
| Normalized Enrichment Score (NES) | 1.4352971 |
| Nominal p-value | 0.06728972 |
| FDR q-value | 0.29124328 |
| FWER p-Value | 0.866 |
Table: GSEA Results Summary

  

Fig 1: Enrichment plot: KEGG\_HUNTINGTONS\_DISEASE      
 Profile of the Running ES Score & Positions of GeneSet Members on the Rank Ordered List

  

| SYMBOL | TITLE | RANK IN GENE LIST | RANK METRIC SCORE | RUNNING ES | CORE ENRICHMENT || 1 | CREB5 | na | 418 | 0.573 | 0.0456 | Yes |
| 2 | TGM2 | na | 541 | 0.536 | 0.0961 | Yes |
| 3 | DNAH2 | na | 1174 | 0.424 | 0.1204 | Yes |
| 4 | COX8C | na | 1278 | 0.411 | 0.1588 | Yes |
| 5 | PLCB1 | na | 1718 | 0.367 | 0.1830 | Yes |
| 6 | PPARG | na | 2303 | 0.323 | 0.1986 | Yes |
| 7 | CLTCL1 | na | 2354 | 0.319 | 0.2293 | Yes |
| 8 | GRM5 | na | 2585 | 0.304 | 0.2532 | Yes |
| 9 | IFT57 | na | 4052 | 0.234 | 0.2344 | Yes |
| 10 | DLG4 | na | 4696 | 0.211 | 0.2371 | Yes |
| 11 | PPARGC1A | na | 6498 | 0.157 | 0.2008 | Yes |
| 12 | ITPR1 | na | 7473 | 0.131 | 0.1858 | Yes |
| 13 | APAF1 | na | 7669 | 0.126 | 0.1929 | Yes |
| 14 | DNAL1 | na | 7730 | 0.125 | 0.2037 | Yes |
| 15 | HAP1 | na | 7988 | 0.119 | 0.2082 | Yes |
| 16 | NDUFA4L2 | na | 8390 | 0.110 | 0.2077 | Yes |
| 17 | NRF1 | na | 8910 | 0.101 | 0.2029 | Yes |
| 18 | GRIN1 | na | 8945 | 0.100 | 0.2120 | Yes |
| 19 | HIP1 | na | 9096 | 0.097 | 0.2174 | Yes |
| 20 | SLC25A4 | na | 9319 | 0.093 | 0.2204 | Yes |
| 21 | SOD2 | na | 9422 | 0.092 | 0.2266 | Yes |
| 22 | AP2A1 | na | 9542 | 0.090 | 0.2322 | Yes |
| 23 | SLC25A31 | na | 9626 | 0.088 | 0.2386 | Yes |
| 24 | CREB1 | na | 9769 | 0.085 | 0.2431 | Yes |
| 25 | SIN3A | na | 9866 | 0.083 | 0.2487 | Yes |
| 26 | TAF4B | na | 10016 | 0.081 | 0.2525 | Yes |
| 27 | EP300 | na | 10138 | 0.079 | 0.2570 | Yes |
| 28 | POLR2K | na | 10875 | 0.068 | 0.2425 | Yes |
| 29 | CLTB | na | 10927 | 0.067 | 0.2478 | Yes |
| 30 | DNALI1 | na | 11136 | 0.064 | 0.2482 | Yes |
| 31 | VDAC1 | na | 11345 | 0.061 | 0.2484 | Yes |
| 32 | CYC1 | na | 11419 | 0.060 | 0.2523 | Yes |
| 33 | SDHB | na | 11751 | 0.056 | 0.2484 | Yes |
| 34 | CREB3L2 | na | 11898 | 0.054 | 0.2496 | Yes |
| 35 | CREB3L4 | na | 12000 | 0.053 | 0.2519 | Yes |
| 36 | TBPL1 | na | 12442 | 0.047 | 0.2439 | Yes |
| 37 | POLR2L | na | 12507 | 0.046 | 0.2467 | Yes |
| 38 | BDNF | na | 12595 | 0.045 | 0.2487 | Yes |
| 39 | BBC3 | na | 12611 | 0.045 | 0.2527 | Yes |
| 40 | COX7B2 | na | 12668 | 0.044 | 0.2555 | Yes |
| 41 | DNAL4 | na | 12914 | 0.041 | 0.2526 | Yes |
| 42 | HDAC2 | na | 13042 | 0.040 | 0.2530 | Yes |
| 43 | SOD1 | na | 13340 | 0.037 | 0.2481 | Yes |
| 44 | UQCRB | na | 13482 | 0.035 | 0.2476 | Yes |
| 45 | NDUFS2 | na | 13540 | 0.035 | 0.2495 | Yes |
| 46 | DCTN4 | na | 13601 | 0.034 | 0.2512 | Yes |
| 47 | NDUFA6 | na | 13652 | 0.034 | 0.2531 | Yes |
| 48 | SLC25A6 | na | 13704 | 0.033 | 0.2550 | Yes |
| 49 | UQCRH | na | 13770 | 0.033 | 0.2564 | Yes |
| 50 | COX7A1 | na | 14036 | 0.030 | 0.2518 | Yes |
| 51 | POLR2E | na | 14079 | 0.030 | 0.2536 | Yes |
| 52 | CASP9 | na | 14196 | 0.029 | 0.2531 | Yes |
| 53 | NDUFV2 | na | 14197 | 0.029 | 0.2560 | Yes |
| 54 | COX4I1 | na | 14252 | 0.028 | 0.2572 | Yes |
| 55 | POLR2D | na | 14385 | 0.027 | 0.2561 | Yes |
| 56 | NDUFA2 | na | 14444 | 0.026 | 0.2571 | Yes |
| 57 | TP53 | na | 14698 | 0.024 | 0.2522 | Yes |
| 58 | AP2B1 | na | 14815 | 0.023 | 0.2511 | Yes |
| 59 | NDUFA4 | na | 14988 | 0.021 | 0.2483 | Yes |
| 60 | TFAM | na | 15039 | 0.021 | 0.2489 | Yes |
| 61 | UQCRFS1 | na | 15042 | 0.021 | 0.2509 | Yes |
| 62 | COX6A1 | na | 15045 | 0.021 | 0.2529 | Yes |
| 63 | NDUFA7 | na | 15138 | 0.020 | 0.2523 | Yes |
| 64 | POLR2J | na | 15173 | 0.020 | 0.2533 | Yes |
| 65 | COX7A2L | na | 15220 | 0.019 | 0.2539 | Yes |
| 66 | NDUFB7 | na | 15235 | 0.019 | 0.2554 | Yes |
| 67 | NDUFA3 | na | 15254 | 0.019 | 0.2568 | Yes |
| 68 | COX4I2 | na | 15306 | 0.018 | 0.2572 | Yes |
| 69 | POLR2G | na | 15364 | 0.018 | 0.2573 | Yes |
| 70 | TAF4 | na | 15463 | 0.017 | 0.2562 | Yes |
| 71 | SDHA | na | 15471 | 0.017 | 0.2577 | Yes |
| 72 | PPID | na | 15617 | 0.016 | 0.2551 | No |
| 73 | NDUFS7 | na | 15694 | 0.015 | 0.2545 | No |
| 74 | DCTN1 | na | 15738 | 0.015 | 0.2547 | No |
| 75 | CREB3 | na | 15750 | 0.015 | 0.2559 | No |
| 76 | SDHD | na | 15796 | 0.014 | 0.2560 | No |
| 77 | POLR2B | na | 15837 | 0.014 | 0.2563 | No |
| 78 | POLR2I | na | 15931 | 0.013 | 0.2549 | No |
| 79 | COX5A | na | 15943 | 0.013 | 0.2560 | No |
| 80 | UQCR10 | na | 16088 | 0.012 | 0.2530 | No |
| 81 | UQCRC2 | na | 16211 | 0.011 | 0.2506 | No |
| 82 | CYCS | na | 16260 | 0.011 | 0.2503 | No |
| 83 | NDUFA9 | na | 16374 | 0.010 | 0.2480 | No |
| 84 | AP2S1 | na | 16461 | 0.009 | 0.2465 | No |
| 85 | COX7C | na | 16469 | 0.009 | 0.2471 | No |
| 86 | POLR2F | na | 16643 | 0.008 | 0.2429 | No |
| 87 | NDUFB4 | na | 16810 | 0.006 | 0.2387 | No |
| 88 | UQCRQ | na | 16876 | 0.006 | 0.2374 | No |
| 89 | VDAC2 | na | 16978 | 0.005 | 0.2350 | No |
| 90 | CREB3L3 | na | 16998 | 0.005 | 0.2349 | No |
| 91 | NDUFB2 | na | 17205 | 0.003 | 0.2292 | No |
| 92 | COX8A | na | 17213 | 0.003 | 0.2292 | No |
| 93 | VDAC3 | na | 17448 | 0.001 | 0.2225 | No |
| 94 | NDUFB6 | na | 17476 | 0.000 | 0.2218 | No |
| 95 | BAX | na | 17508 | 0.000 | 0.2209 | No |
| 96 | POLR2J3 | na | 17578 | -0.000 | 0.2189 | No |
| 97 | NDUFB3 | na | 17733 | -0.002 | 0.2147 | No |
| 98 | NDUFB9 | na | 17840 | -0.003 | 0.2119 | No |
| 99 | DCTN2 | na | 17872 | -0.003 | 0.2113 | No |
| 100 | NDUFA5 | na | 17888 | -0.003 | 0.2111 | No |
| 101 | NDUFC1 | na | 17903 | -0.003 | 0.2110 | No |
| 102 | COX6C | na | 17968 | -0.004 | 0.2096 | No |
| 103 | NDUFS8 | na | 18032 | -0.004 | 0.2082 | No |
| 104 | GPX1 | na | 18080 | -0.005 | 0.2073 | No |
| 105 | COX7A2 | na | 18090 | -0.005 | 0.2075 | No |
| 106 | SLC25A5 | na | 18293 | -0.007 | 0.2023 | No |
| 107 | NDUFB10 | na | 18344 | -0.007 | 0.2016 | No |
| 108 | AP2M1 | na | 18393 | -0.007 | 0.2009 | No |
| 109 | UQCRC1 | na | 18411 | -0.008 | 0.2012 | No |
| 110 | NDUFA8 | na | 18467 | -0.008 | 0.2005 | No |
| 111 | NDUFS3 | na | 18481 | -0.008 | 0.2009 | No |
| 112 | POLR2A | na | 18527 | -0.009 | 0.2005 | No |
| 113 | COX5B | na | 18689 | -0.010 | 0.1969 | No |
| 114 | NDUFB8 | na | 18767 | -0.011 | 0.1957 | No |
| 115 | NDUFS4 | na | 18768 | -0.011 | 0.1968 | No |
| 116 | HDAC1 | na | 19008 | -0.013 | 0.1912 | No |
| 117 | COX6B1 | na | 19017 | -0.013 | 0.1923 | No |
| 118 | TBP | na | 19024 | -0.013 | 0.1934 | No |
| 119 | CREB3L1 | na | 19199 | -0.015 | 0.1899 | No |
| 120 | NDUFAB1 | na | 19284 | -0.015 | 0.1890 | No |
| 121 | SDHC | na | 19318 | -0.016 | 0.1896 | No |
| 122 | COX7B | na | 19335 | -0.016 | 0.1907 | No |
| 123 | UQCR11 | na | 19468 | -0.017 | 0.1886 | No |
| 124 | NDUFV3 | na | 19559 | -0.018 | 0.1878 | No |
| 125 | POLR2C | na | 19762 | -0.019 | 0.1839 | No |
| 126 | CLTA | na | 19774 | -0.020 | 0.1855 | No |
| 127 | NDUFS5 | na | 19842 | -0.020 | 0.1856 | No |
| 128 | NDUFA1 | na | 19847 | -0.020 | 0.1876 | No |
| 129 | REST | na | 19849 | -0.020 | 0.1896 | No |
| 130 | DNAI2 | na | 19863 | -0.020 | 0.1913 | No |
| 131 | NDUFV1 | na | 19894 | -0.021 | 0.1925 | No |
| 132 | NDUFC2 | na | 19991 | -0.022 | 0.1919 | No |
| 133 | NDUFB1 | na | 20064 | -0.022 | 0.1921 | No |
| 134 | GNAQ | na | 20613 | -0.028 | 0.1790 | No |
| 135 | PLCB4 | na | 20753 | -0.029 | 0.1779 | No |
| 136 | POLR2H | na | 20975 | -0.031 | 0.1747 | No |
| 137 | UCP1 | na | 21041 | -0.032 | 0.1760 | No |
| 138 | NDUFS6 | na | 21061 | -0.032 | 0.1787 | No |
| 139 | RCOR1 | na | 21534 | -0.038 | 0.1689 | No |
| 140 | POLR2J2 | na | 21612 | -0.038 | 0.1705 | No |
| 141 | NDUFB5 | na | 22347 | -0.047 | 0.1540 | No |
| 142 | NDUFS1 | na | 22850 | -0.053 | 0.1448 | No |
| 143 | NDUFA10 | na | 23223 | -0.058 | 0.1398 | No |
| 144 | CASP8 | na | 23310 | -0.059 | 0.1433 | No |
| 145 | PLCB3 | na | 24121 | -0.071 | 0.1271 | No |
| 146 | COX6B2 | na | 24380 | -0.075 | 0.1272 | No |
| 147 | SP1 | na | 24777 | -0.082 | 0.1240 | No |
| 148 | CASP3 | na | 24828 | -0.083 | 0.1308 | No |
| 149 | CREBBP | na | 24831 | -0.083 | 0.1391 | No |
| 150 | DNAI1 | na | 25670 | -0.098 | 0.1246 | No |
| 151 | DNAH1 | na | 26235 | -0.109 | 0.1193 | No |
| 152 | HTT | na | 26490 | -0.115 | 0.1235 | No |
| 153 | AP2A2 | na | 27123 | -0.128 | 0.1182 | No |
| 154 | GRIN2B | na | 28274 | -0.158 | 0.1008 | No |
| 155 | PLCB2 | na | 28336 | -0.160 | 0.1152 | No |
| 156 | TBPL2 | na | 29675 | -0.200 | 0.0966 | No |
| 157 | COX6A2 | na | 30724 | -0.237 | 0.0902 | No |
| 158 | CLTC | na | 31200 | -0.254 | 0.1021 | No |
Table: GSEA details [plain text format]

  

Fig 2: KEGG\_HUNTINGTONS\_DISEASE      
 Blue-Pink O' Gram in the Space of the Analyzed GeneSet

  

Fig 3: KEGG\_HUNTINGTONS\_DISEASE: Random ES distribution      
 Gene set null distribution of ES for **KEGG\_HUNTINGTONS\_DISEASE**
